# Health, Work, and Social Problems in Spanish Informal Caregivers: Does Gender Matter? (The CUIDAR-SE Study)

**DOI:** 10.3390/ijerph18147332

**Published:** 2021-07-08

**Authors:** Luz María Peña-Longobardo, María Del Río-Lozano, Juan Oliva-Moreno, Isabel Larrañaga-Padilla, María del Mar García-Calvente

**Affiliations:** 1 Departamento de Análisis Económico y Seminario de Investigación en Economía y Salud (SIES), Universidad de Castilla-La Mancha, 45071 Toledo, Spain; LuzMaria.Pena@uclm.es (L.M.P.-L.); Juan.OlivaMoreno@uclm.es (J.O.-M.); 2 Escuela Andaluza de Salud Pública (EASP), 18080 Granada, Spain; mariadelmar.garcia.easp@juntadeandalucia.es; 3 Instituto de Investigación Biosanitaria de Granada ibs. Granada, 18012 Granada, Spain; 4 Departamento de Salud del Gobierno Vasco, Delegación de Salud de Gipuzkoa, 20010 San Sebastián, Spain; mlarranagapadilla@gmail.com

**Keywords:** informal care, caregiver, gender differences, health problems, work problems, social problems

## Abstract

(1) Background: The aim of this study was (i) to analyze problems faced by informal caregivers in three areas of their life: health, work and finances, and family and social relationships, (ii) to investigate the main determinants of these problems, and (iii) to explore differences between men and women. (2) Methods: The study population consisted of people aged ≥18 years living in a family home who were providing unpaid care to a dependent person in the same or another home and who were registered as caregivers with the Primary Health Care District of Granada or the Provincial Council of Gipuzkoa. Several logistic regression models were built to analyze the likelihood of caregivers experiencing health, work-related, or social problems as a result of their caregiving responsibilities. (3) Results: Informal female caregivers were more likely to experience problems attributed to caregiving than their male counterparts, particularly in the areas of health and work. Additional factors associated with an increased likelihood of problems were low perceived social support, performance of ungratifying tasks, and fewer years as a caregiver. (4) Conclusions: Informal caregivers in Spain face significant problems as a result of their caregiving duties, and the impact on men and women is different. Policies and interventions to mitigate the negative effects of unpaid caregiving should incorporate differential strategies to meet the specific needs of male and female caregivers in different caregiving contexts.

## 1. Introduction

Informal care, which is unpaid care provided by family members, friends, or neighbors, is the main source of care for dependent persons [1]. The nature of this care varies considerably from country to country, both in terms of the support systems in place and the proportion of the population providing informal care, ranging from around 13% in countries, such as Spain, to more than 19% in countries, such as Finland. However, the intensity of caregiving is generally higher in southern Europe than in northern Europe. Over 30% of informal care in Mediterranean countries (and 50% of that in Spain) is high-intensity care, that is, the caregiver carries out more than 20 h per week of caregiving [2]. The requirement for greater dedication and availability can lead caregivers to often feel a loss of control over their time, and this can negatively affect different aspects of their life.

There is evidence that the strain and demands of caregiving have a detrimental effect on the health and well-being of caregivers, particularly for those providing intense long-term care [3,4]. Physical and psychological health effects are the most widely studied effects of caregiving in Spain [5,6] and other countries [7,8]. Some authors have highlighted the high opportunity costs associated with caregiving, including missed family, social, and leisure opportunities (due to a lack of time [9,10]). More mental health problems, such as stress, depression, and emotional distress, have also been reported for caregivers versus non-caregivers. Caregiver distress is especially aggravated in care situations, such as intense care over 21 h weekly, caring for someone with dementia, depression, or responsive behaviors and lives with the care receiver [11,12,13]. Informal caregivers have an increased risk of losing their jobs or missing career advancement opportunities. In fact, studies from different countries have demonstrated a negative association between caregiving and participation in the labor force [14,15,16,17,18,19,20].

Regarding gender, most informal caregivers in Europe are women, especially in countries from the south and center of Europe. They spend more time than men caring for others and more often have to make significant changes to their lives to take on this role [21,22]. Although there is ample literature on the range of problems facing caregivers, few studies have analyzed the role of gender [23]. While it is recognized that women continue to shoulder the bulk of informal care duties, there is an increasing trend of men, and retired men in particular, taking more responsibility in this area; this trend is expected to increase as the population ages [24,25,26]. Research must therefore reflect this new situation and include men alongside the dominant group of female caregivers to explore this emerging profile and investigate gender-based differences in caregiving and its consequences.

The aim of this study was to analyze problems faced by informal male and female caregivers from two regions of Spain in three areas of their life: health, work and finances, and social and family relationships. We also explored the main determinants of the problems identified and analyzed differences between men and women.

## 2. Materials and Methods

### 2.1. Data

Data were collected in a cross-sectional interview-based study conducted within the framework of the CUIDAR-SE study, which analyzed health-related quality of life (HRQoL) among informal male and female caregivers in the Andalusian province of Granada in southern Spain and the Basque province of Gipuzkoa in northern Spain. The study population consisted of adults (≥18 years) living in a family home who were providing informal (unpaid) care to a dependent person in the same or another home and who were registered as caregivers with the Primary Health Care District of Granada or enrolled in the social services dependency registry of the Provincial Council of Gipuzkoa created following the Spanish Dependency Law (DL) [27]. The DL, launched in 2007, gave rise to the current System of Autonomy and Care for Dependency, which is the set of services and benefits aimed at promoting personal autonomy, as well as the protection and care of people, through accredited public and private concerted services. These benefits are granted in all cases to the dependent person in all Spanish regions [28].

The caregivers selected to participate in the study were identified using a three-stage cluster random sampling approach in which municipalities were established as primary units, census sectors within these municipalities as secondary units, and caregivers as final units. Municipalities were stratified by size and caregivers by gender. The study received ethical approval by the Research Ethics Committee of Granada and informed consent was obtained from all participants. The study and methodology are described in detail elsewhere [29,30].

In sample selection, we did not seek a sample in which the proportion of male and female caregivers was representative of the population. We sought to sample a sufficient and equivalent number of women and men to be able to compare different elements related to care. Due to the selection method chosen, most of the caregivers were classified as high-intensity caregivers.

### 2.2. Outcome Indicators

The main outcome indicators (problems attributed to caregiving by the interviewees), were the following (in all of them the response categories were yes or no):

- Health problems: (i) general health problems (any of the problems in categories ii to vi), (ii) deterioration of health, (iii) need for treatment, (iv) tiredness, (v) feelings of depression, and (vi) other health-related problems.
- Work or financial problems: (i) general work or financial problems (any of the problems in categories ii to vi), (ii) unable to perform paid work (already quit or cannot consider working outside the home), (iii) problems meeting work schedules, and (iv) financial/economic difficulties.
- Social or family problems: (i) general social or family problems (any of the problems in categories ii to vi) (ii) less time for social activities/no holidays, (iii) no time for self-care or to care for others, (iv) no time to meet up with friends, (v) deterioration in relationship with care recipient, and (vi) deterioration in relationship with family and/or partner.

The explanatory variables considered were: (1) caregiver characteristics, for instance, gender, age (older ≥65 years, middle 50–64 years, and young <50 years), level of education (no education completed, primary, secondary, and tertiary level), place of residence (Granada and Gipuzkoa), household income adjusted by household size and composition according to the OECD-modified scale, classified into three groups: low (<EUR 1000 a month), average (EUR 1000–1500 a month), and high (>EUR 1500 a month), and health-related quality of life (HRQoL) using the EQ-5D-5L index, which ranges from 0 to 1, were dichotomized into high HRQoL (score > 0.85) and low HRQoL (score ≤ 0.85); (2) caregiving characteristics, for instance, years spent providing care, and performance of ungratifying personal care tasks (where participants responded yes or no for assistance with bathing or showering, washing or toileting, diapering for urinary incontinence, or diapering for fecal incontinence); (3) perceived social support, through the abbreviated Duke Social Support Index with 11 items validated for use in the Spanish population [31], dichotomized into high and low; and (4) use of health and social care services by care recipient or caregiver, for instance, services in home, such as home help, services outside the home, such as respite services, allowances, such as PECF and other benefits, and other types of support services, such as information, training, or psychological support. The general health status of the care recipient was also considered through the self-perception of the caregiver.

### 2.3. Method

Numerous logistic regression models were built to analyze the odds of caregivers experiencing problems related to health, work and finances, and social and family relationships. Dependent variables were assigned a value of 1 if the caregiver identified a problem and 0 otherwise.

The general structure of the model was as follows:

$$prob_{i}\left(problemj\right)\;=\;\wedge \left(\alpha_{1}-\beta \prime_{1}X_{i}-\varepsilon_{i}\right)$$

$$prob_{i}\left(problemj\right)\;=\;\wedge \left(\alpha_{j}-\beta \prime_{j}X_{i}\right)-\wedge \left(\alpha_{j-1}-\beta \prime_{j-1}X_{i}\right)-\varepsilon_{i},$$

$$j=2,\;\ldots ,\;j-1prob_{i}\left(problemj\right)=1-\overset{j-1}{\underset{j=1}{\sum }}prob_{i}\left(problemj\right)$$

where probi (problemj) is the likelihood that the caregiver *i* (*i* = 1, …, I) perceives a given problem where *j* = 1, 0; Ʌ denotes the logistic distribution function; and *X_i_* represents the vector of the explanatory variables—caregiver age, gender, level of education, place of residence, adjusted household income, HRQoL, self-perceived health of the care recipient high perceived social support, performance of ungratifying tasks, receipt of allowances, and use of health and social care services in the home and outside the home—; *β* is the vector of the coefficient parameters assigned to each explanatory variable included in vector *X*; and *ε_i_* is the standard error.

The extended model is as follows:

*Prob (problem attributed to caregiving for each dimension) = β0 + β1 (middle-older age) + β2 (older age) + β3 (years providing care) + β4 (female) + β5 (primary education) + β6 (secondary/tertiary-level education) + β7 (middle adjusted household income) + β8 (high adjusted household income) + β9 (Granada) + β10 (high HRQoL) + β11 (self-perceived health of the care recipient) + β12 (performance of ungratifying tasks) + β13 (high perceived social support) + β14 (use of health and social care services in the home) + β15 (use of health and social care services outside the home) + β16 (receipt of allowances) + β17 (use of other formal services) + ut.*

We built 16 logistic regression models: one for each category within the three dimensions (health, work/finances, social/family relationships). All the models were additionally stratified by gender.

## 3. Results

The main sociodemographic characteristics of the study population and their use of health and social care services are summarized in Table 1. Compared with women, male caregivers were older, had been providing care for less time, and were less likely to benefit from allowances or other formal support services. The proportions of male and female caregivers who perceived specific problems within the three dimensions (health, work/finances, and social/family relationships) are shown in Table 2. Overall, women had more problems because of their caregiving responsibilities in all the dimensions.

In the multivariate analysis (Table 3), women were more likely than men to have health problems (OR: 2.69) and work or financial (OR: 2.33) problems. Place of residence (Granada), low perceived social support, performance of ungratifying tasks, and poor perceived care recipient health were significantly associated with a higher likelihood of problems in general. Additional determinants of problems in this dimension were number of years providing care and age. In both cases, the likelihood decreased with number of years and age. The only significant determinant of work problems (in caregivers aged <65 years) was living in Granada. Gender was not a significant determinant of social or family problems. Having a secondary or tertiary-level education, by contrast, significantly increased the odds of a problem in this dimension (OR: 2.23).

The results stratified by gender are shown in Table 3. Place of residence was significantly associated with perceived problems in all dimensions for women and in the health and work or finances dimensions for men. Likewise, women who had to perform ungratifying personal care tasks and who perceived strong support from their social networks were more likely to have health problems (OR: 3.13 and OR: 0.16, respectively). These variables were not significant in the other two dimensions. More nuanced results were observed within each dimension. High perceived social support was a clear protective factor for men, and was statistically significant in all three dimensions. Men who had to perform ungratifying tasks were significantly more likely to have problems with their health (OR: 4.24) and with social and family relationships (OR: 3.08).

Within the health dimension, gender was a significant determinant of deteriorated health (OR: 1.61), need for treatment (OR: 2.54), tiredness (OR: 3.30), and feelings of depression (OR: 1.84). The only health category in which women were less likely than men to experience problems was “other health-related problems” (OR: 0.62) (see Table S1, Supplementary Materials). The results for the health dimension categories stratified by gender are shown in Table 4. Caregiver HRQoL was a significant determinant of several health problems for both men and women. Additional predictors were place of residence, performance of ungratifying tasks, and perceived social support.

Women were more likely than men to report that they were unable to perform paid work because of their caregiving responsibilities (OR: 3.61) and equally likely to mention difficulties meeting work schedules or financial difficulties (Table S2, Supplementary Materials). The results for perceived work-related problems (caregivers < 65 years of age) and financial difficulties (all caregivers) are shown in Table 5. Living in Granada as opposed to Gipuzkoa was associated with a greater likelihood of work or financial problems among both male and female caregivers. The odds of not being able to do paid work because of caregiving duties were high in Granada. Perceived care recipient health and household income were also identified as significant determinants of paid work or financial problems, although gender differences were observed in certain categories. Caregiver age was significant for women only. In the case of men, years spent providing care, level of education, and HRQoL were significant determinants of some of the problems analyzed. Women, but not men, who perceived strong support from their social networks were significantly more likely to report not being able to perform paid work because of caregiving.

The only problems perceived by women in the social and family relationships dimension were no time to meet friends (OR: 1.54), and no time to care for themselves or others (OR: 1.57), although this second variable was not significant (Table S3, Supplementary Materials). The results, broken down by categories and stratified by gender, are summarized in Table 6. Caregivers who had to perform ungratifying tasks were significantly more likely to have less time for social activities, including meeting up with friends, be unlikely to take holidays, and look after themselves and others. Caregiver HRQoL was also a significant explanatory variable in this dimension, but with differences between men and women. Caregivers who perceived strong support from their social networks were significantly less likely to report deterioration in their relationship with the care recipient and with their family and/or partner. Other significant determinants of problems in the social and family relationship dimension were place of residence and perceived health of the person being cared for. In the case of men, years spent providing care was associated with a lower likelihood of problems in several categories in this dimension.

## 4. Discussion

This study provides knowledge on a wide variety of problems associated with informal care, and offers a gender-based analysis of the differences between women and men. These two strengths of the analysis have allowed the results to be especially novel compared to what is already known in this field. The main finding of this study is that informal female caregivers in the north and south of Spain are more likely than their male counterparts to experience problems as a result of caregiving, particularly in the areas of health and work. This may partly be because women perceive the demands of caregiving more intensely than men [32,33]. In fact, the literature has consistently shown that greater caregiving burden among women is associated with lower societal recognition of the value of their work and deep-seated gender norms that leave them with little freedom to make decisions on what their role should be [34]. These social expectations mean that women experience the pressure of caregiving more than men and this has a greater impact on their health and other aspects of their life [35].

One of the main determinants of problems attributed to caregiving in our series was social support. Strong support from one’s social networks was associated with a lower likelihood of problems in the three dimensions (health, work/finances, and social/family relationships) for men and in the health dimension for women. This is consistent with the literature, which has shown that social support is an important predictor of population health [36,37] and can help alleviate stress or make problems seem smaller [38,39]. A recent study found that women mainly received help from women with a similar profile, while men had broader, more diverse social networks and received more help from outside the family circle [40]. The means by which men and women sought specific help also varied. While women generally sought less help and relied more on support from family members than on formal support or paid help, men made greater use of formal services and shared their caregiving responsibilities with more people [30]. The lower levels of formal support received by women could be linked to the higher prevalence of health, professional, economic, and personal problems attributed to caregiving among female caregivers [41].

Numbers of years spent caregiving was also a significant explanatory variable, but only for men, who were less likely to experience problems with social or family relationships when they had been providing care for longer. This could be due to the “adaptation effect”, by which a given effect loses intensity as the person adapts to a new situation over time [42,43,44]. Number of years providing care was not a significant determinant of problems in any of the dimensions for women, possibly because they are better able to react to sudden changes to their situation as they have traditionally been assigned the role of caregiver and frequently view this role as natural or as a moral obligation [45].

The nature of care provided is an important consideration when analyzing the impact of caregiving from a gender perspective. In our series, caregivers who had to perform ungratifying tasks, such as changing diapers or providing personal care and hygiene assistance, were more likely to experience problems with their health (men and women) or with social or family relationships (men). The odds ratios of men experiencing problems in these areas, however, were particularly high, possibly because male caregivers have been found to take more responsibility for gratifying tasks and to delegate more burdensome tasks to others before their health is seriously affected [35].

Place of residence was a determinant of problems attributed to caregiving. Caregivers in Granada were more likely than those from Gipuzkoa to have problems in all the dimensions studied. The differences were most pronounced in the work dimension and were particularly evident among male caregivers. One explanation could be the origin of the samples. In Gipuzkoa, the caregivers were identified through the social services registry of dependent persons of the Provincial Council. The registry was created under the Spanish DL, which established that dependent persons may benefit from various in-kind benefits and services, depending on their level of dependency and the availability of services from the competent institution. In Gipuzkoa, the dependents included in the registry received a cash-for-care allowance (PECF)—financial benefit for care in the family setting. In Granada, however, the caregivers were identified through health registries, meaning that the person they were caring for may or may not have been receiving this allowance. Financial support may have mitigated the negative effects of caregiving in the case of Gipuzkoa, as it has been shown that monetary interventions can have a moderating effect on caregiving burden [46]. Socioeconomic differences between the two regions and in the support systems in place may also have had an effect. Implementation of the DL varies considerably from one region to the next as it has been hampered by several challenges that emerged in the context of the recent economic crisis [28]. The PECF, for example, is received by 34% of dependent persons in Andalusia compared with 52% of those in the Basque Country [47]. The lower coverage rates in Andalusia could explain the higher prevalence of problems attributed to caregiving in Granada, as a combination of formal and informal care has been found to counteract some of the negative impacts of caregiving [48,49]. The labor market is also different in the north and south of Spain. The unemployment rate in Granada (south Spain) in 2020 was 24%, for example, compared with 8% in Gipuzkoa (north Spain) [50]. In fact, greater difficulties finding and keeping a job in Granada could explain why male caregivers in this province perceived more work problems in relation to caregiving, particularly considering the dominant role that paid employment has traditionally played in the construction of male identity [51]. The above aspects highlight the complex relationships between care recipients, caregivers, and caregiving context. More studies are needed to analyze the macro and micro factors that influence caregiving.

This study has some limitations. First, as it is a cross-sectional study, we cannot draw any causal links between the problems identified and the variables analyzed. Nonetheless, this limitation was partly overcome by asking the caregivers about “problems due to caregiving”. Second, our findings cannot be extrapolated to Spain as a whole, as we studied just two regions. One advantage of this approach, however, is that it adds a richness to our analysis as Granada and Gipuzkoa differ both socioeconomically and in terms of coverage of services for dependent people. One particular strength of our study is that we did not focus on specific diseases, as some recent studies have done [52], but on care recipients with a wide range of needs. A high proportion of our study sample (78%) benefits from the PECF. This differentiates this sample from the general population of unpaid caregivers in Spain. In December 2020 there were 1,385,037 recognized dependents (valued by the system established by the Dependency Law), of which 83% receive some type of provision or service by the Law, and 33% receive the PECF [53]. This benefit has an average monthly amount of EUR 306 and, although the monetary value is clearly insufficient to offset the costs of care, it can help to alleviate some of its consequences on caregivers. We believe that studying the problems involved in caring for this specific group of caregivers can be a strength of the study and contribute new knowledge to the subject. Finally, we only analyzed registered caregivers. Nevertheless, we believe that people who are not registered probably dedicate less time to caregiving. The profile of caregiver in our study thus is that of a male or female caregiver providing long-term high-intensity care. Our findings could, therefore, be extrapolated to caregivers with a similar profile, whom we believe should be prioritized in support interventions.

Our findings indicate that intense care involving the performance of ungratifying tasks in a context with little informal or formal support can further deteriorate the health of both male and female caregivers. Improvements to policies and formal support services are urgently needed to help women and men providing unpaid care to dependent relatives. Thus, the results obtained show that informal caregivers in Spain face significant problems in different areas of their life as a consequence of caregiving, as well as highlights considerable differences between men and women. Policies and interventions to mitigate the negative impacts of informal caregiving should therefore incorporate differential strategies to meet the specific needs of male and female caregivers in different caregiving contexts. Other authors have identified needs-based strategies and a more equitable distribution of caregiving resources as essential tools for reducing gender inequalities in health [33,54]. To achieve this, it is necessary to find ways of ensuring the visibility of caregiving and of increasing the social recognition of the work carried out by informal caregivers. Policies promoting a fairer distribution of care work among men and women and among all social agents involved are also needed.

The recent global health crisis caused by COVID-19 has brought to the foreground the crucial role of informal caregivers and the significant burden they often have to shoulder [55]. In addition, a number of recent studies have shown that caregiving during the pandemic has increased the risk of exposure and infection among women [56,57]. Similarly, the increase in in-home caregiving as a result of the pandemic could exacerbate the unequal distribution of gender roles, further aggravating health inequalities [58]. Although some countries have implemented specific measures to support informal caregivers during the pandemic [59], more formal resources are needed, as are gender-based policies addressing the specific needs of men and women providing care [60]. Our study confirms that informal male and female caregivers have different profiles and needs, and as they experience problems differently, these differences need to be taken into account when designing policies and support interventions. The current social, economic, and health crisis has made even more evident the need to continue investigating inequalities in caregiving and to incorporate gender considerations into this research.

## 5. Conclusions

Informal caregivers in Spain face significant problems as a consequence of caregiving, but the impact on women and men is varies greatly. In the analyzed setting, female caregivers were more likely to experience problems as a result of their caregiving duties, particularly in the dimensions of health and paid work. Policies and interventions to mitigate these effects should incorporate differential strategies addressing the specific needs of men and women in different caregiving contexts.

## Figures and Tables

**Table 1:** Sociodemographic characteristics of informal caregivers.

	Total (n = 610)	Male (n = 265)	Female (n = 345)	Comparison of Means *p*-Value
	Average (SD) or %	Average (SD) or %	Average (SD) or %	
Gender (female)	56.56	-	-	-
Age (mean, SD)	59.82 (14.47)	62.28 (16.28)	57.94 (12.62)	0.0002 **
Years spent caregiving (mean, SD)	9.40 (8.54)	7.96 (0.46)	10.49 (0.50)	0.0004 **
Education				
No education completed	40.07	45.08	36.23	0.1841
Primary Education	25.94	21.21	29.57	
Secondary/tertiary-level education	33.99	33.71	34.20	
Household income (mean, SD)	1157.59 (539.99)	1212.51 (34.87)	1113.87 (30.21)	0.0324 *
Caregiver HRQoL (mean, SD)	0.827 (0.194)	0.836 (0.203)	0.821 (0.187)	0.3232
Poor care recipient health as perceived by caregiver	35.15	32.95	36.81	0.3250
Living in Granada	51.31	50.19	52.17	0.6275
High perceived social support (yes)	80.16	76.98	82.60	0.0843 *
Ungratifying tasks (yes)	51.15	48.30	53.33	0.2185
Health and social Services				
Services at home	85.74	84.15	86.96	0.3268
Services outside the home	17.38	13.96	20.00	0.0512
Monetary benefits	79.51	73.96	83.77	0.0029 **
Other services	66.56	66.04	66.96	0.8119
Relationship of the cared-for person to the caregiver				0.0000 **
Spouses/partner	37.70	55.09	24.35	
Daughter/son	12.79	5.66	18.26	
Mother/father	40.16	32.08	46.38	
Mother/father in-law	1.64	1.51	1.74	
Other relatives	7.71	5.66	9.28	

HRQoL, health-related quality of life. * Statistically significant at 95%; ** statistically significant at 99%.

**Table 2:** Problems attributed to caregiving by male and female caregivers in three dimensions: health, work/finances, and social/family relationships.

	Total (n = 610)	Male (n = 265)	Female (n = 345)	Comparison of Means *p*-Value
**Health problems (%)**				
General (any of below)	67.70	58.49	74.79	0.0000 **
Deteriorated health	38.69	31.32	44.34	0.0010 **
Needs treatment	16.72	10.18	21.73	0.0001 **
Feels tired	51.48	37.35	62.31	0.0000 **
Feels depressed	26.72	21.13	31.01	0.0062 **
Other health-related problems	10.49	12.45	8.98	0.1665
**Work/financial problems ^1^ (%)**				
General problems (any of below)	67.06	58.19	72.01	0.0092 **
Cannot work	40.29	28.68	46.78	0.0010 **
Difficulty meeting work schedules	26.76	29.50	25.22	0.3942
Financial difficulties	47.59	51.47	45.37	0.2576
**Social/family relationship problems (%)**				
General problems (any of below)	80.16	75.84	83.48	0.0191 *
Less time for social activities/no holidays	53.95	50.18	56.85	0.1025
No time for self-care or to care for others	41.54	36.36	45.50	0.0233 *
No time to see friends	56.60	50.95	60.93	0.0140 *
Deterioration in relationship with care recipient	8.42	6.92	9.58	0.2478
Deterioration in relationship with family and/or partner	5.75	4.15	6.97	0.1379

^1^ Only caregivers <65 years old. * Statistically significant at 95%. ** Statistically significant at 99%.

**Table 3:** Multivariate analysis of problems attributed to caregiving by full sample and men and women separately.

	All			Women			Men		
	Health	Work and Finances	Social and Family	Health	Work and Finances	Social and Family	Health	Work and Finances	Social and Family
	Odds Ratio (SE)	Odds Ratio (SE)	Odds Ratio (SE)	Odds Ratio (SE)	Odds Ratio (SE)	Odds Ratio (SE)	Odds Ratio (SE)	Odds Ratio (SE)	Odds Ratio
Female	2.694 ** (0.673)	2.328 * (0.785)	1.442 (0.385)	----	----	----	----	----	----
Age (50–64)	0.492 * (0.156)	1.380 (0.474)	0.965 (0.351)	0.388 * (0.176)	1.057 (0.461)	0.492 (0.272)	0.654 (0.322)	3.084 (2.113)	2.372 (1.415)
Age (≥65 years)	0.724 (0.254)	----	0.741 (0.294)	0.814 (0.481)	----	0.514 (0.336)	0.635 (0.328)	----	1.132 (0.649)
Years spent providing care	0.971 ** (0.014)	0.978 (0.027)	0.988 (0.014)	0.959 (0.020)	0.988 (0.033)	0.986 (0.021)	0.960 (0.022)	0.988 (0.060)	0.983 (0.023)
Primary education	0.711 (0.233)	0.564 (0.338)	1.303 (0.461)	0.306 * (0.156)	0.602 (0.461)	1.590 (0.843)	1.294 (0.631)	0.683 (0.850)	0.818 (0.434)
Secondary/tertiary-level education	1.565 (0.520)	0.586 (0.345)	2.229 * (0.821)	1.570 (0.829)	0.332 (0.240)	2.439 (1.319)	1.903 (0.934)	2.822 (3.722)	2.698 (1.529)
Average adjusted monthly household income (€1000–1500)	1.325 (0.374)	1.029 (0.415)	0.943 (0.281)	0.954 (0.424)	0.946 (0.509)	0.576 (0.264)	1.707 (0.674)	1.347 (1.003)	1.566 (0.694)
High adjusted monthly household income (>€1500)	1.328 (0.448)	0.601 (0.272)	1.170 (0.436)	1.290 (0.670)	0.641 (0.385	1.050 (0.583)	1.340 (0.666)	0.249 (0.230)	1.075 (0.606)
Living in Granada	3.170 ** (0.929)	12.578 ** (5.546)	3.060 ** (1.014)	4.651 ** (2.227)	6.331 ** (3.632)	5.403 ** (2.994)	2.642 * (1.098)	119.060 ** (128.95)	1.419 (0.673)
Caregiver HRQoL (high)	0.175 ** (0.053)	1.428 (0.553)	0.364 ** (0.123)	0.108 ** (0.057)	1.111 (0.550)	0.327 * (0.173)	0.210 ** (0.085)	5.446 (4.790)	0.343 * (0.167)
Poor care recipient health as perceived by caregiver	2.051 ** (0.497)	1.709 (0.586)	2.017 ** (0.532)	3.208 ** (1.202)	2.087 (0.949)	2.346 * (0.986)	1.292 (0.631)	2.545 (1.695)	1.714 (0.648)
Ungratifying tasks	3.539 ** (0.873)	0.827 (0.276)	2.230 ** (0.603)	3.126 ** (1.227)	0.525 (0.244)	1.855 (0.776)	4.235 ** (1.471)	2.135 (1.369)	3.077 ** (1.259)
High perceived social support	0.301 ** (0.114)	0.407 (0.194)	0.288 * (0.145)	0.160 * (0.135)	0.599 (0.402)	0.434 (0.353)	0.313 * (0.147)	0.170 * (0.150)	0.200 * (0.132)
Health and social care services at home	1.022 (0.329)	0.651 (0.307)	1.156 (0.396)	0.550 (0.295)	0.589 (0.396)	0.541 (0.333)	1.568 (0.711)	1.163 (0.933)	2.733 (1.318)
Health and social care services outside the home	1.521 (0.493)	1.131 (0.463)	1.530 (0.564)	2.649 (1.363)	0.881 (0.460)	2.395 (1.382)	1.025 (0.479)	2.775 (2.401)	0.833 (0.451)
Allowances	1.061 (0.369)	0.897 (0.489)	1.618 (0.607)	1.177 (0.859)	0.275 (0.302)	0.818 (0.712)	1.057 (0.442)	2.190 (2.077)	2.022 (0.928)
Other services	0.916 (0.250)	0.748 (0.285)	0.803 (0.235)	1.577 (0.683)	0.912 (0.469)	0.852 (0.380)	0.656 (0.257)	0.334 (0.237)	0.823 (0.352)
N	529	293	529	296	187	296	233	106	233
LR chi^2^	190.24	97.91	93.04	130.16	49.67	71.29	72.24	59.66	44.23
Pseudo R^2^	0.2877	0.2803	0.1846	0.3860	0.2493	0.2751	0.2311	0.4218	0.1825

HRQoL, health-related quality of life; SE, standard error. * Statistically significant at 95%. ** Statistically significant at 99%. Omitted variable: younger than 50 years old.

**Table 4:** Analysis of health problems perceived by male and female caregivers.

	Deteriorated Health		Needs Treatment		Feels Tired		Feels Depressed		Other Health-Related Problem	
	Women	Men	Women	Men	Women	Men	Women	Men	Women	Men
	Odds Ratio (SE)	Odds Ratio (SE)	Odds Ratio (SE)	Odds Ratio (SE)	Odds Ratio (SE)	Odds Ratio (SE)	Odds Ratio (SE)	Odds Ratio (SE)	Odds Ratio (SE)	Odds Ratio (SE)
Age (50–64)	0.990 (0.374)	0.979 (0.489)	0.915 (0.397)	0.569 (0.446)	0.870 (0.313)	0.974 (0.449)	0.628 (0.235)	0.803 (0.440)	0.574 (0.300)	1.623 (0.970)
Age (≥65 years)	0.997 (0.479)	0.575 (0.323)	1.012 (0.545)	0.228 (0.205)	0.817 (0.375)	1.131 (0.566)	0.449 (0.216)	0.733 (0.456)	0.591 (0.406)	1.256 (0.886)
Years spent providing care	1.035 (0.019)	1.002 (0.025)	0.990 (0.019)	1.020 (0.035)	0.964 (0.016)	0.976 (0.022)	0.969 (0.018)	0.986 (0.026)	1.009 (0.024)	0.968 (0.035)
Primary education	1.226 (0.496)	2.300 (1.155)	1.002 (0.458)	2.805 (2.535)	0.545 (0.217)	1.392 (0.648)	0.654 (0.265)	2.047 (1.136)	0.832 (0.501)	0.833 (0.547)
Secondary/tertiary-level education	1.751 (0.714)	2.276 (0.686)	1.814 (0.811)	6.562 * (5.931)	1.108 (0.443)	1.516 (0.713)	0.962 (0.385)	1.747 (1.063)	1.110 (0.667)	2.270 (1.431)
Average adjusted monthly household income (€1000–1500)	0.802 (0.295)	2.226 * (0.904)	1.394 (0.534)	2.303 (1.458)	0.904 (0.313)	1.839 (0.683)	1.361 (0.486)	1.219 (0.522)	0.570 (0.286)	1.023 (0.509)
High adjusted monthly household income (>EUR 1500)	2.342 (1.084)	1.316 (0.737)	0.708 (0.388)	1.072 (1.023)	1.230 (0.525)	1.898 (0.901)	1.583 (0.723)	0.863 (0.524)	0.254 (0.186)	0.788 (0.498)
Living in Granada	3.825 ** (1.487)	2.898 * (1.278)	0.922 (0.402)	9.382 (8.055)	3.516 ** (1.308)	1.330 (0.524)	0.990 (0.383)	2.622 (1.315)	0.317 * (0.181)	2.110 (1.134)
Caregiver HRQoL (high)	0.200 ** (0.065)	0.178 ** (0.067)	0.146 ** (0.054)	0.204 * (0.127)	0.353 ** (0.118)	0.226 ** (0.077)	0.214 ** (0.068)	0.195 (0.078)	0.504 (0.236)	1.610 (0.784)
Poor care recipient health as perceived by caregiver	2.272 * (0.777)	3.632 ** (1.512)	1.983 (0.844)	0.759 (0.500)	1.380 (0.4369	1.707 (0.584)	2.197 * (0.789)	1.442 (0.640)	1.843 (0.977)	0.952 (0.434)
Ungratifying tasks	2.532 ** (0.846)	2.606 ** (0.935)	1.908 (0.720)	0.627 (0.357)	2.081 * (0.641)	1.274 (0.400)	0.880 (0.2889	2.008 (0.784)	1.662 (0.794)	3.690 ** (1.717)
High perceived social support	0.251 ** (0.108)	0.638 (0.259)	0.572 (0.227)	0.207 ** (0.121)	0.245 ** (0.122)	0.471 * (0.182)	0.334 ** (0.122)	0.401 * (0.169)	3.592 (2.845)	0.879 (0.461)
Health and social care services at home	0.801 (0.402)	0.831 (0.469)	0.678 (0.389)	2.230 (2.443)	0.353 (0.162)	0.968 (0.449)	0.500 (0.252)	1.006 (0.678)	1.140 (0.854)	2.186 (1.556)
Health and social care services outside the home	1.693 (0.659)	1.377 (0.685)	3.065 ** (1.284)	0.782 (0.718)	1.920 (0.767)	2.085 (0.922)	2.516 (0.975)	0.679 (0.402)	1.056 (0.601)	0.400 (0.280)
Allowances	1.029 (0.453)	1.223 (0.506)	1.212 (0.531)	0.870 (0.548)	1.661 (0.780)	0.681 (0.256)	0.854 (0.339)	1.219 (0.528)	0.838 (0.539)	0.975 (0.497)
Other services	0.813 (0.322)	1.278 (0.579)	1.063 (0.449)	0.341 (0.284)	1.998 (0.718)	0.986 (0.380)	2.090 (0.831)	0.888 (0.459)	1.085 (0.595)	0.393 (0.205)
N	296	233	296	233	296	233	296	233	296	233
LR chi^2^	122.71	78.04	62.36	50.27	88.76	51.59	70.72	50.81	13.45	17.27
Pseudo R^2^	0.3005	0.2627	0.2018	0.3348	0.2260	0.1642	0.1928	0.2097	0.0744	0.0965

HRQoL, health-related quality of life; SE, standard error. * Statistically significant at 95%. ** Statistically significant at 99%. Omitted variable: younger than 50 years old.

**Table 5:** Analysis of work and financial problems perceived by male and female caregivers.

	Cannot Work ^1^		Difficulty Meeting Work Schedules ^1^		Financial Difficulties	
	Women	Men	Women	Men	Women	Men
	Odds Ratio (SE)	Odds Ratio (SE)	Odds Ratio (SE)	Odds Ratio (SE)	Odds Ratio (SE)	Odds Ratio (SE)
Age (50–64)	2.832 * (1.364)	0.997 (0.781)	0.447 (0.191)	1.750 (1.016)	0.504 * (0.168)	0.731 (0.339)
Age (≥65 years)	-----	-----	-----	-----	0.770 (0.319)	0.396 (0.200)
Years spent providing care	0.977 (0.031)	0.810 * (0.067)	1.001 (0.031)	1.088 (0.053)	1.005 (0.015)	0.958 (0.024)
Primary education	0.889 (0.589)	0.704 (0.673)	1.203 (0.759)	0.930 (0.792)	1.587 (0.560)	1.644 (0.777)
Secondary/tertiary-level education	0.320 (0.194)	0.119 * (0.129)	1.444 (0.839)	4.968 (4.166)	1.970 (0.702)	1.673 (0.829)
Average adjusted monthly household income (EUR 1000–1500)	0.196 ** (0.100)	6.898 * (6.626)	4.512 ** (2.160)	0.777 (0.463)	1.102 (0.344)	1.040 (0.384)
High adjusted monthly household income (>EUR 1500)	0.183 * (0.129)	9.067 (11.048)	3.0977 (1.857)	0.378 (0.288)	0.702 (0.291)	0.882 (0.428)
Living in Granada	15.727 ** (9.377)	716.421 ** (1230.183)	0.386 (0.210)	1.477 (0.944)	2.460 ** (0.830)	2.342 * (0.951)
Caregiver HRQoL (high)	1.320 (0.591)	0.858 (0.679)	0.795 (0.351)	1.875 (1.180)	0.716 (0.207)	0.465 * (0.168)
Poor care recipient health as perceived by caregiver	2.836 * (1.342)	4.133 (3.420)	0.966 (0.425)	0.956 (0.538)	1.975 * (0.603)	2.982 * (0.951)
Ungratifying tasks	1.075 (0.504)	1.004 (0.799)	0.596 (0.253)	1.515 (0.799)	1.093 (0.316)	3.680 ** (1.232)
High perceived social support	0.157 ** (0.109)	0.200 (0.169)	3.308 (2.257)	1.243 (0.752)	1.017 (0.360)	0.503 (0.202)
Health and social care services at home	0.231 * (0.162)	0.363 (0.610)	1.359 (0.817)	2.146 (1.664)	0.354 * (0.157)	0.560 (0.263)
Health and social care services outside the home	0.773 (0.441)	0.750 (0.795)	1.079 (0.549)	2.064 (1.362)	1.348 (0.468)	0.571 (0.264)
Allowances	2.082 (1.296)	13.697 ** (13.044)	0.241 ** (0.139)	0.349 (0.216)	1.113 (0.420)	1.029 (0.399)
Other services	1.079 (0.637)	0.254 (0.357)	1.043 (0.516)	0.591 (0.363)	1.471 (0.5100)	0.998 (0.397)
N	187	106	187	106	293	232
LR chi^2^	112.33	74.12	39.99	19.17	47.13	66.31
Pseudo R^2^	0.4340	0.5572	0.1896	0.1497	0.1167	0.2094

^1^ Only considered caregivers younger than 65 years old. HRQoL, health-related quality of life; SE, standard error. * Statistically significant at 95%. ** Statistically significant at 99%. Omitted variable: younger than 50 years old.

**Table 6:** Analysis of problems with social and family relationships perceived by male and female caregivers.

	Less Time for Social Activities/No Holidays		No Time for Self-Care or to Care for Others		No Time to See Friends		Deterioration in Relationship with Care Recipient		Deterioration in Relationship with Family/Partner	
	Women	Men	Women	Men	Women	Men	Women	Men	Women	Men
	Odds Ratio (SE)	Odds Ratio (SE)	Odds Ratio (SE)	Odds Ratio (SE)	Odds Ratio (SE)	Odds Ratio (SE)	Odds Ratio (SE)	Odds Ratio (SE)	Odds Ratio (SE)	Odds Ratio (SE)
Age (50–64)	1.261 (0.411)	1.265 (0.578)	0.879 (0.319)	1.581 (0.810)	1.035 (0.367)	1.976 (0.950)	2.829 (2.036)	0.304 (0.278)	1.304 (0.825)	0.295 (0.303)
Age (≥65 years)	1.340 (0.554)	0.966 (0.469)	0.829 (0.384)	1.001 (0.556)	0.761 (0.336)	1.703 (0.867)	4.124 (3.375)	0.147 (0.160)	0.683 (0.606)	0.039 * (0.060)
Years spent providing care	0.985 (0.015)	0.953 * (0.022)	0.988 (0.017)	0.936 * (0.024)	0.995 (0.016)	0.948 * (0.023)	1.027 (0.023)	0.975 (0.046)	1.017 (0.031)	0.946 (0.063)
Primary education	0.910 (0.318)	0.778 (0.352)	0.761 (0.300)	1.090 (0.560)	1.137 (0.421)	1.082 (0.514)	1.732 (1.118)	0.997 (0.916)	1.059 (0.870)	0.270 (0.333)
Secondary/tertiary-level education	1.409 (0.499)	1.649 (0.768)	1.148 (0.454)	1.567 (0.858)	2.556 * (1.005)	2.165 (1.072)	2.213 (1.387)	0.310 (0.358)	2.624 (1.870)	0.141 (0.206)
Average adjusted monthly household income (EUR 1000–1500)	0.993 (0.306)	0.968 (0.350)	0.719 (0.253)	0.702 (0.278)	1.074 (0.364)	1.460 (0.564)	1.374 (0.722)	0.249 (0.235)	1.238 (0.785)	0.078 (0.106)
High adjusted monthly household income (>EUR 1500)	1.677 (0.669)	0.952 (0.442)	1.049 (0.472)	0.468 (0.248)	1.752 (0.742)	0.656 (0.318)	1.116 (0.793)	3.554 (3.401)	1.820 (1.443)	0.472 (0.702)
Living in Granada	1.762 (0.591)	2.112 (0.823)	4.473 ** (1.672)	9.721 ** (4.565)	4.549 ** (1.669)	2.618 * (1.054)	0.620 (0.360)	0.018 ** (0.023)	1.133 (0.831)	0.361 (0.404)
Caregiver HRQoL (high)	0.659 (0.192)	0.448 * (0.161)	0.352 ** (0.112)	0.640 (0.237)	0.501 * (0.160)	0.344 ** (0.128)	1.035 (0.503)	0.029 ** (0.029)	0.988 (0.569)	0.112 ** (0.118)
Poor care recipient health as perceived by caregiver	1.606 (0.466)	2.590 ** (0.864)	2.226 * (0.724)	2.719 * (1.066)	1.632 (0.510)	1.274 (0.436)	2.163 (1.262)	2.403 (2.046)	1.317 (0.869)	--------
Ungratifying tasks	1.916 * (0.540)	3.100 ** (1.004)	3.038 ** (0.976)	3.111 ** (1.127)	1.280 (0.387)	3.235 ** (1.096)	1.871 (0.966)	0.205 (0.166)	3.007 (1.868)	1.128 (0.944)
High perceived social support	0.473 (0.183)	0.396 (0.162)	0.522 (0.215)	0.254 (0.110)	0.704 (0.290)	0.151 ** (0.072)	0.116 ** (0.059)	0.091 ** (0.074)	0.083 ** (0.049)	0.174 * (0.139)
Health and social care services at home	0.641 (0.268)	1.291 (0.581)	0.301 * (0.143)	0.475 (0.248)	0.636 (0.291)	1.258 (0.579)	2.101 (1.787)	0.185 (0.170)	0.930 (0.725)	0.122 (0.180)
Health and social care services outside the home	1.126 (0.388)	0.416 (0.185)	2.055 (0.815)	1.433 (0.731)	1.856 (0.712)	0.645 (0.290)	1.457 (0.77)	2.821 (2.982)	1.468 (0.886)	3.329 (3.774)
Allowances	0.948 (0.383)	2.254 * (0.867)	0.834 (0.367)	1.361 (0.556)	1.037 (0.465)	1.825 (0.739)	0.992 (0.626)	0.172 * (0.153)	0.386 (0.254)	0.565 (0.533)
Other services	1.138 (0.370)	0.766 (0.288)	1.128 (0.431)	0.366 (0.164)	0.752 (0.268)	0.777 (0.299)	0.513 (0.266)	1.050 (0.860)	0.223 * (0.140)	1.667 (1.994)
N	295	233	296	232	295	231	287	228	296	158
LR chi^2^	38.78	56.48	112.99	88.97	63.81	72.61	32.79	47.01	37.03	26.16
Pseudo R^2^	0.0969	0.1751	0.2756	0.2863	0.1651	0.2274	0.1787	0.3886	0.2363	0.3277

HRQoL, health-related quality of life; SE, standard error. * Statistically significant at 95%. ** Statistically significant at 99%.

## Data Availability

Data belong to the survey of CUIDAR-SE and the data are owned by the Escuela Andaluza de Salud Pública and, hence, under the Escuela Andaluza‘regulations as it contains potentially sensitive information on individuals. People interested in data viewing can contact María del Mar García Calvente.

## Supplementary Materials

The following are available online at https://www.mdpi.com/article/10.3390/ijerph18147332/s1, Table S1: Analysis of health problems (full sample), Table S2: Analysis of work and financial problems (all sample), Table S3: Analysis of problems with social and family relationships (all sample).

## References

[1] Colombo F., Llena-Nozal A., Mercier J., Tjadens F. (2011). Help Wanted? Providing and Paying for Long-Term Care.

[2] OECD (2019). Care Needed: Improving the Lives of People with Dementia.

[3] Legg L., Weir C.J., Langhorne P. (2013). Is informal caregiving independently associated with poor health? A population-based study. J. Epidemiol. Community Health.

[4] Smith L., Onwumere J., Craig T. (2014). Mental and physical illness in caregivers: Results from an English national survey sample. Br. J. Psychiatry.

[5] García-Calvente M.M., Mateo-Rodríguez I., Maroto-Navarro G. (2004). El impacto de cuidar en la salud y la calidad de vida de las mujeres. Gac. Sanit..

[6] Larrañaga I., Martín U., Bacigalupe A., Begiristáin J.M., Valderrama M.J., Arregi B. (2008). Impacto del cuidado informal en la salud y la calidad de vida de las personas cuidadoras: Análisis de las desigualdades de género. Gac. Sanit..

[7] Pinquart M., Sörensen S. (2007). Correlates of Physical Health of Informal Caregivers: A Meta-Analysis. B Psychol. Sci. Soc. Sci..

[8] Vitaliano P.P., Zhang J., Scanlan J.M. (2003). Is caregiving hazardous to one’s physical health? A meta-analysis. Psychol. Bull..

[9] Bittman M., Fast E., Fisher K., Thomson C., Bittman M., Folbre N. (2004). Making the Invisible Visible: The Life and Time(s) of Informal Caregivers. Family Time: The Social Organisation of Care.

[10] Rodríguez-Madrid M.N., del Río-Lozano M., Fernández-Peña R., Elizalde B., García-Calvente M.M. (2020). Redes personales de apoyo y cuidado informal: ¿Diferencias por sexo y territorio? (estudio CUIDAR-SE II). Gac. Sanit..

[11] Pinquart M., Sörensen S. (2003). Differences between caregivers and noncaregivers in psychological health and physical health: A meta-analysis. Psychol. Aging.

[12] Roth D.L., Perkins M., Wadley V.G., Temple E.M., Haley W.E. (2009). Family caregiving and emotional strain: Associations with quality of life in a large national sample of middle-aged and older adults. Qual. Life Res..

[13] Schulz R., Sherwood P. (2008). Physical and mental health effects of family caregiving. Am. J. Nurs..

[14] Bolin K., Lindgren B., Lundborg P. (2008). Your next of kin or your own career? Caring and working among the 50+ of Europe. J. Health Econ..

[15] Fast J., Dosman D., Lero D., Lucas S. (2013). The Intersection of Caregiving and Employment across the Life Course.

[16] Plaisier I., Broese M.I., Keuzenkamp S. (2015). Combining work and informal care: The importance of caring organisations. Hum. Resour. Manag. J..

[17] Masuy A.J. (2009). Effect of caring for an older person on women’s lifetime participation in work. Ageing Soc..

[18] Lilly M.B., Laporte A., Coyte P.C. (2007). Labor market work and home care’s unpaid caregivers: A systematic review of labor force participation rates, predictors of labor market withdrawal, and hours of work. Milbank Q..

[19] Heitmueller A., Michaud P.C. (2006). Informal Care and Employment in England: Evidence from the British Household Panel Survey.

[20] García-Calvente M.M., del Río-Lozano M. (2010). Análisis del Impacto de Cuidar Sobre la Salud de Mujeres y Hombres.

[21] Verbakel E., Tamlagsrønning S., Winstone L., Fjær E.L., Eikemo T.A. (2017). Informal care in Europe: Findings from the European Social Survey (2014) special module on the social determinants of health. Eur. J. Public Health.

[22] European Institute for Gender Equality (EIGE) (2021). Gender Inequalities in Care and Consequences for the Labour Market. Beijing Platform for Action.

[23] Lee Y., Xu L., Kim B.J., Chen L. (2019). Leisure activity, gender and depressive symptoms among dementia caregivers: Findings from the REACH II. Aging Ment. Health.

[24] Esparza C. (2011). Discapacidad y Dependencia en España.

[25] Abellán A., Ayala A., Pérez J., Pujol R. (2018). Los Nuevos Cuidadores. https://observatoriosociallacaixa.org/-/los-nuevos-cuidadores.

[26] Sundström G., Jegermalm M., Abellán A., Ayala A., Pérez J., Pujol R., Souto J. (2018). Men and older persons also care, but how much? Assessing amounts of caregiving in Spain and Sweden. Int. J. Ageing Later Life.

[27] Ley 39/2006, de 14 de Diciembre, de Promoción de la Autonomía Personal y Atención a las Personas en Situación de Dependencia (Promotion of Personal Autonomy and Care for Dependent People Act). https://www.boe.es/eli/es/l/2006/12/14/39/con.

[28] Peña-Longobardo L.M., Oliva-Moreno J., García-Armesto S., Hernández-Quevedo C. (2016). The Spanish long-term care system in transition: Ten years since the 2006 Dependency Act. Health Policy.

[29] Oliva-Moreno J., Peña-Longobardo L.M., García-Mochón L., Del Río-Lozano M., Mosquera-Metcalfe I., García-Calvente M.M. (2019). The economic value of time of informal care and its determinants (The CUIDARSE Study). PLoS ONE.

[30] Del Río-Lozano M., García-Calvente M.M., Calle-Romero J., Machón-Sobrado M., Larrañaga-Padilla I. (2017). Health-related quality of life in Spanish informal caregivers: Gender differences and support received. Qual. Life Res..

[31] Bellón-Saameño J.A., Delgado-Sánchez A., Luna-del Castillo J.D., Lardelli-Claret P. (1996). Validez y fiabilidad del cuestionario de apoyo social funcional Duke-UNC-11. Aten. Primaria.

[32] García-Mochón L., Peña-Longobardo L.M., del Río-Lozano M., Oliva-Moreno J., Larrañaga-Padilla I., García-Calvente M.M. (2019). Determinants of burden and satisfaction in informal caregivers: Two sides of the same coin? The CUIDAR-SE study. Int. J. Environ. Res. Public Health.

[33] Adelman R.D., Tmanova L.L., Delgado D., Dion S., Lachs M.S. (2014). Caregiver burden: A clinical review. JAMA.

[34] Cascella-Carbó G.F., García-Orellán R. (2020). Burden and Gender inequalities around Informal Care. Investig. Educ. Enferm..

[35] Del Río-Lozano M., García-Calvente M.M., Marcos-Marcos J., Entrena-Durán F., Maroto-Navarro G. (2013). Gender identity in informal care: Impact on health in Spanish caregivers. Qual. Health Res..

[36] Matud M.P., García M.C., Fortes D. (2019). Relevance of Gender and Social Support in Self-Rated Health and Life Satisfaction in Elderly Spanish People. Int. J. Environ. Res. Public Health.

[37] Ergh T.C., Rapport L.J., Coleman R.D., Hanks R.A. (2002). Predictors of Caregiver and Family Functioning Following Traumatic Brain Injury: Social Support Moderates Caregiver Distress. J. Head Trauma Rehabil..

[38] Mohanty I., Niyonsenga T., Cochrane T., Rickwood D. (2020). A multilevel mixed effects analysis of informal carers health in Australia: The role of community participation, social support and trust at small area level. BMC Public Health.

[39] Cohen C.A., Colantonio A., Vernich L. (2002). Positive aspects of caregiving: Rounding out the caregiver experience. Int. J. Geriatr. Psychiatry.

[40] Rodríguez-Madrid M.N., del Río-Lozano M., Fernandez-Peña R., Jiménez-Pernett J., García-Mochón L., Lupiañez-Castillo A., García-Calvente M.M. (2018). Gender Differences in Social Support Received by Informal Caregivers: A Personal Network Analysis Approach. Int. J. Environ. Res. Public Health.

[41] Abajo M., Rodríguez-Sanz M., Malmusi D., Salvador M., Borrell C. (2017). Gender and socio-economic inequalities in health and living conditions among co-resident informal caregivers: A nationwide survey in Spain. J. Adv. Nurs..

[42] Infurna F.J., Wiest M., Gerstorf D., Ram N., Schupp J., Wagner G.G., Heckhausen J. (2017). Changes in life satisfaction when losing one’s spouse: Individual differences in anticipation, reaction, adaptation and longevity in the German Socio-economic Panel Study (SOEP). Ageing Soc..

[43] Kahneman D., Thaler R. (1991). Economic analysis and the psychology of utility: Applications to compensation policy. Am. Econ. Rev..

[44] Easterlin R.A. (2005). A puzzle for adaptive theory. J. Econ. Behav. Organ..

[45] Gilligan C. (1985). La Moral y la Teoría.

[46] Leszko M. (2019). The Effectiveness of Psychoeducational and Financial Intervention to Support Caregivers of Individuals with Alzheimer’s Disease in Poland. Innov. Aging.

[47] (2019). Círculo Empresarial de Atención a las Personas (CEAPS). https://www.acra.cat/informe-el-caos-de-la-atención-a-la-dependencia-una-perspectiva-desde-la-gestión-_464277.pdf.

[48] Van den Broek T., Grundy E. (2020). Does long-term care coverage shape the impact of informal care-giving on quality of life? A difference-in-difference approach. Ageing Soc..

[49] Bass D.M., Noelker L.S., Rechlin L.R. (1996). The Moderating Influence of Service Use on Negative Caregiving Consequences. B Psychol. Sci. Soc. Sci..

[50] Instituto Nacional de Estadística. https://www.ine.es/.

[51] Marcos-Marcos J., Mateos J.T., Gasch-Gallén À., Álvarez-Dardet C. (2020). The study of health in men from a gender perspective: Where we come from, where are we going. Salud Colect..

[52] Li Q.P., Mak Y.W., Loke A.Y. (2013). Spouses’ experience of caregiving for cancer patients: A literature review. Int. Nurs. Rev..

[53] Ramírez-Navarro J.M., Revilla-Castro A., Fuentes-Jiménez M., Gómez-Castro E. (2021). XXI Dictamen del Observatorio Estatal de la Dependencia.

[54] Palència L., de Moortel D., Artazcoz L., Salvador-Piedrafita M., Puig-Barrachina V., Hagqvist E., Pérez G., Ruiz M.E., Trujillo-Alemán S., Vanroelen C. (2017). Gender Policies and Gender Inequalities in Health in Europe: Results of the SOPHIE Project. Int. J. Health Serv..

[55] Lorenz-Dant K., Comas-Herrera A. (2021). The Impacts of COVID-19 on Unpaid Carers of Adults with Long-Term Care Needs and Measures to Address These Impacts: A Rapid Review of the Available Evidence up to November 2020. J. Long Term Care.

[56] Wenham C., Smith J., Morgan R., Gender and COVID-19 Working Group (2020). COVID-19: The gendered impacts of the outbreak. Lancet.

[57] Castellanos-Torres E., Tomás-Mateos J., Chilet-Rosell E. (2020). COVID-19 en clave de género. Gac. Sanit..

[58] Del Río-Lozano M., García-Calvente M.M. (2020). Grupo de alumnado del Diploma de Especialización en Género y Salud de la Escuela Andaluza de Salud Pública-Universidad de Granada. Cuidados y abordaje de la pandemia de COVID-19 con enfoque de género. Gac. Sanit..

[59] Lorenz-Dant K. (2020). International examples of measures to support unpaid carers during the COVID-19 pandemic Report in LTCcovid.org, International Long-Term Care Policy Network, CPEC-LSE. https://ltccovid.org/2020/05/20/new-report-international-examples-of-measures-to-support-unpaid-carers-during-the-covid-19-pandemic/.

[60] ONU Mujeres Las Mujeres y el COVID-19: Cinco Acciones que los Gobiernos Pueden Adoptar sin Demoras. https://www.unwomen.org/es/news/stories/2020/3/news-women-and-covid-19-governments-actions-by-ded-bhatia.

