# Supplementary material for: Health, Work, and Social Problems in Spanish Informal Caregivers: Does Gender Matter? (The CUIDAR-SE Study)
Source: Int J Environ Res Public Health. 2021 Jul 8;18(14):7332. doi: 10.3390/ijerph18147332 (PMC8306791; doi:10.3390/ijerph18147332)
Supplement: Supplementary file 1 [file ijerph-18-07332-s001.zip › ijerph-1262339-supplementary.pdf]

Supplementary Materials

Table S1. Analysis of health problems (full sample)

	**Deteriorated health**		**Needs treatment**		**Feels tired**		**Feels depressed**		**Other health-related problem**	
	**Odds Ratio (SE)**	**P value**	**Odds Ratio (SE)**	**P value**	**Odds Ratio (SE)**	**P value**	**Odds Ratio (SE)**	**P value**	**Odds Ratio (SE)**	**P value**
Women	**1.611 (0.377)**	**0.041**	**2.536 (0.774)**	**0.002**	**3.301 (0.735)**	**0.000**	**1.839 (0.448)**	**0.013**	**0.615 (0.187)**	**0.112**
Age (50-64)	0.950 (0.278)	0.861	0.721 (0.255)	0.356	0.919 (0.250)	0.759	0.690 (0.206)	0.216	0.956 (0.361)	0.906
Age (≥65 years)	0.718 (0.250)	0.344	0.614 (0.266)	0.262	0.953 (0.305)	0.883	**0.494 (0.179)**	**0.052**	**0.884 (0.408)**	**0.790**
Years spent providing care	1.021 (0.014)	0.137	0.995 (0.016)	0.802	0.969 (0.012)	0.018	**0.978 (0.014)**	**0.153**	**0.999 (0.019)**	**0.969**
Primary education	1.583 (0.488)	0.136	1.319 (0.509)	0.474	0.898 (0.258)	0.711	0.949 (0.300)	0.870	0.969 (0.417)	0.943
Secondary/ third-level education	1.455 (0.454)	0.229	**2.176 (0.833)**	**0.042**	1.287 (0.373)	0.383	1.038 (0.334)	0.905	1.511 (0.633)	0.324
Average adjusted monthly household income (€1000–€1500)	1.296 (0.344)	0.329	1.568 (0.493)	0.153	1.257 (0.312)	0.356	1.284 (0.346)	0.354	0.769 (0.263)	0.445
High adjusted monthly household income (>€1500)	1.648 (0.556)	0.139	0.954 (0.425)	0.917	1.487 (0.453)	0.192	1.437 (0.497)	0.294	0.608 (0.270)	0.264
Living in Granada	**2.982 (0.830)**	**0.000**	1.645 (0.594)	0.168	**2.286 (0.592)**	**0.001**	1.348 (0.396)	0.309	0.936 (0.303)	0.839
Caregiver HRQoL (high)	**0.197 (0.046)**	**0.000**	**0.178 (0.053)**	**0.000**	**0.298 (0.069)**	**0.000**	**0.219 (0.053)**	**0.000**	0.936 (0.303)	0.839
Poor care recipient health as perceived by caregiver	**2.668 (0.675)**	**0.000**	1.697 (0.576)	0.119	**1.583 (0.355)**	**0.040**	**1.799 (0.487)**	**0.030**	1.183 (0.393)	0.612
Ungratifying tasks	**2.334 (0.545)**	**0.000**	1.473 (0.432)	0.186	**1.583 (0.336)**	**0.030**	1.203 (0.287)	0.437	**2.349 (0.743)**	**0.007**
High perceived social support	**0.426 (0.117)**	**0.002**	**0.374 (0.114)**	**0.001**	**0.429 (0.121)**	**0.003**	**0.354 (0.094)**	**0.000**	1.312 (0.520)	0.493
Health and social care services at home	0.854 (0.307)	0.661	0.786 (0.362)	0.602	0.609 (0.190)	0.113	0.684 (0.259)	0.319	1.532 (0.754)	0.387
Health and social care services outside the home	1.711 (0.506)	0.069	**2.066 (0.712)**	**0.035**	**1.877 (0.526)**	**0.025**	1.531 (0.457)	0.154	0.650 (0.272)	0.304
Allowances	1.093 (0.319)	0.760	1.137 (0.386)	0.705	0.911 (0.263)	0.748	0.942 (0.271)	0.837	0.930 (0.356)	0.851
Other services	**0.961 (0.174)**	**0.041**	0.864 (0.306)	0.681	1.378 (0.348)	0.204	1.429 (0.428)	0.234	0.701 (0.249)	0.318
N	529		529		529		529		529	
LR chi2	192.70		100.89		146.30		113.90		15.74	
Pseudo R2	0.2699		0.2133		0.1999		0.1851		0.0435	

Table S2. Analysis of work and financial problems (all sample)

	Cannot work		Difficulty meeting work schedules		Financial difficulties	
	**Odds Ratio (SE)**	**P value**	**Odds Ratio (SE)**	**P value**	**Odds Ratio (SE)**	**P value**
Woman	**3.611 (1.410)**	**0.001**	0.841 (0.258)	0.574	1.033 (0.212)	0.873
Age (50-64)	2.224 (0.841)	0.035	0.695 (0.220)	0.251	**0.569 (0.149)**	**0.031**
Age (≥65 years)	-----	----	-----	----	**0.528 (0.160)**	**0.035**
Years spent providing care	0.961 (0.026)	0.153	1.010 (0.023)	0.574	1.000 (0.122)	0.957
Primary education	0.900 (0.454)	0.836	1.025 (0.494)	0.958	**1.717 (0.469)**	**0.048**
Secondary/ third-level education	0.289 (0.141)	0.011	2.104 (0.947)	0.098	**1.734 (0.481)**	**0.047**
Average adjusted monthly household income (€1000–€1500)	**0.469 (0.189)**	**0.061**	2.206 (0.758)	0.021	1.078 (0.249)	0.743
High adjusted monthly household income (>€1500)	0.396 (0.216)	0.089	1.792 (0.763)	0.171	0.793 (0.235)	0.435
Living in Granada	**24.569 (12.352)**	**0.000**	0.715 (0.281)	0.394	**2.186 (0.540)**	**0.002**
Caregiver HRQoL (high)	1.207 (0.436)	0.601	1.046 (0.346)	0.890	**0.645 (0.139)**	**0.043**
Poor care recipient health as perceived by caregiver	**2.505 (0.952)**	**0.016**	0.839 (0.264)	0.580	**2.125 (0.469)**	**0.001**
Ungratifying tasks	1.087 (0.402)	0.821	0.879 (0.265)	0.671	**1.824 (0.375)**	**0.003**
High perceived social support	0.185 (0.085)	0.000	2.026 (0.758)	0.076	0.730 (0.183)	0.212
Health and social care services at home	0.231 (0.139)	0.015	1.474 (0.652)	0.380	**0.463 (0.139)**	**0.011**
Health and social care services outside the home	0.720 (0.335)	0.482	1.156 (0.422)	0.691	0.960 (0.254)	0.878
Allowances	**3.463 (1.571)**	**0.006**	**0.313 (0.122)**	**0.003**	0.996 (0.258)	0.989
Other services	1.002 (0.495)	0.995	0.848 (0.304)	0.647	1.183 (0.291)	0.495
N	293		293		525	
LR chi2	176.71		35.53		88.93	
Pseudo R2	0.4382		0.1046		0.1234	

Table S3. Analysis of problems with social and family relationships (all sample)

	Less time for social activities/no holidays		No time for self-care or to care for others		No time to see friends		Deterioration in relationship with care recipient		Deterioration in relationship with family/partner	
	**Odds Ratio (SE)**	**P value**	**Odds Ratio (SE)**	**P value**	**Odds Ratio (SE)**	**P value**	**Odds Ratio (SE)**	**P value**	**Odds Ratio (SE)**	**P value**
Woman	1.395 (0.286)	0.104	1.572 (0.336)	0.050	**1.541 (0.328)**	**0.042**	1.415 (0.517)	0.342	1.668 (0.736)	0.246
Age (50-64)	1.232 (0.320)	0.423	1.076 (0.309)	0.797	1.218 (0.331)	0.467	1.171 (0.546)	0.735	0.840 (0.408)	0.721
Age (≥65 years)	1.077 (0.325)	0.805	0.814 (0.276)	0.546	0.897 (0.282)	0.731	1.243 (0.681)	0.691	0.376 (0.255)	0.149
Years spent providing care	0.979 (0.011)	0.084	0.972 (0.013)	0.051	0.986 (0.012)	0.294	1.019 (0.019)	0.297	0.996 (0.025)	0.896
Primary education	0.869 (0.236)	0.607	0.866 (0.261)	0.635	1.059 (0.300)	0.837	1.593 (0.794)	0.350	0.865 (0.538)	0.816
Secondary/ third-level education	1.352 (0.371)	0.271	1.123 (0.346)	0.706	**1.942 (0.569)**	**0.023**	1.772 (0.889)	0.254	1.727 (0.967)	0.328
Average adjusted monthly household income (€1000–€1500)	0.985 (0.226)	0.949	0.748 (0.192)	0.259	1.263 (0.307)	0.337	1.167 (0.483)	0.708	0.696 (0.348)	0.470
High adjusted monthly household income (>€1500)	1.271 (0.367)	0.405	0.796 (0.260)	0.487	1.201 (0.358)	0.538	1.266 (0.641)	0.641	0.823 (0.518)	0.758
Living in Granada	**1.780 (0.434)**	**0.018**	**5.888 (1.653)**	**0.000**	**3.599 (0.931)**	**0.000**	**0.371 (0.170)**	**0.031**	1.033 (0.573)	0.952
Caregiver HRQoL (high)	**0.595 (0.130)**	**0.018**	**0.480 (0.112)**	**0.002**	**0.449 (0.104)**	**0.001**	**0.461 (0.175)**	**0.042**	**0.664 (0.286)**	**0.344**
Poor care recipient health as perceived by caregiver	**1.921 (0.403)**	**0.002**	**2.512 (0.610)**	**0.000**	1.384 (0.303)	0.139	2.100 (0.910)	0.087	2.370 (1.300)	0.116
Ungratifying tasks	**2.208 (0.447)**	**0.000**	**2.978 (0.688)**	**0.000**	**1.901 (0.402)**	**0.002**	1.125 (0.411)	0.747	1.474 (0.650)	0.379
High perceived social support	**0.437 (0.117)**	**0.002**	**0.397 (0.113)**	**0.001**	**0.367 (0.108)**	**0.001**	**0.142 (0.054)**	**0.000**	**0.143 (0.062)**	**0.000**
Health and social care services at home	0.867 (0.253)	0.626	**0.395 (0.131)**	**0.005**	0.849 (0.255)	0.588	0.764 (0.390)	0.600	0.703 (0.418)	0.554
Health and social care services outside the home	0.827 (0.216)	0.469	1.737 (0.521)	0.066	1.246 (0.342)	0.422	1.899 (0.791)	0.124	2.373 (1.141)	0.072
Allowances	1.376 (0.365)	0.229	1.042 (0.298)	0.885	1.391 (0.395)	0.244	0.672 (0.304)	0.382	**0.370 (0.183)**	**0.045**
Other services	0.949 (0.224)	0.827	0.694 (0.190)	0.185	0.733 (0.182)	0.212	0.629 (0.257)	0.258	0.556 (0.270)	0.227
N	528		528		526		515		529	
LR chi2	83.44		193.07		115.13		48.67		51.55	
Pseudo R2	0.1151		0.2663		0.1618		0.1594		0.2087	
